# Clinical Characteristics and Risk Factors for Severity and Prognosis of Convulsive Status Epilepticus

**DOI:** 10.1002/brb3.70681

**Published:** 2025-08-21

**Authors:** Zhen Tian, Yingchun Xu, Rundong He, Di Huang, Wanyu Liu, Peiwen Li, Jiasheng Yang, Jie Yang, Jing Ding, Wei Yue

**Affiliations:** ^1^ Tianjin Huanhu Hospital Tianjin China

**Keywords:** clinical characteristics, convulsive status epilepticus, prognosis, risk factors, severity

## Abstract

**Background:**

Convulsive status epilepticus (CSE) is a frequently encountered serious condition in the field of neurology. Research on CSE has always been a key issue in the field of neurological emergencies. Quickly assessing the severity and prognosis of CSE is crucial for guiding treatment. However, the associated risk factors are not yet fully understood. This study aims to examine the clinical characteristics of CSE patients admitted to our hospital and identify the risk factors that influence the severity of CSE and its prognosis.

**Methods:**

We retrospectively collected clinical data from CSE patients admitted to our hospital between July 2023 and December 2024. We grouped patients based on the modified Status Epilepticus Severity Score (mSTESS score) at onset and the Glasgow Outcome Scale score (GOS score) at discharge. This grouping allowed us to assess the severity and prognosis of CSE. We collected clinical characteristics such as age, gender, medical history, etiology, mSTESS score, treatment, comorbidities, duration of seizure, maintenance of antiepileptic drugs at discharge, and GOS score at discharge. The clinical characteristics of CSE patients were analyzed, and related risk factors for severity and prognosis were explored.

**Results:**

A total of 104 patients with CSE were included, consisting of 60 males and 44 females. The average age of patients was 58.05 (±17.50), ranging from 15 to 88 years. Among these patients, generalized convulsive status epilepticus (GCSE) accounted for 76 cases (73.1%), with stroke being the most common cause of CSE, contributing to 45 cases (43.3%). Eight (7.7%) developed into RSE. Severity, defined as mSTESS ≥ 4, was observed in 65 patients (62.5%), while poor outcomes, indicated by GOS ≤ 3, were noted in 40 patients (38.5%). Multivariate logistic analysis demonstrated that the duration of CSE had a statistically significant impact on severity (mSTESS ≥ 4) (95% CI, 1.06–2.47; *p *< 0.05). Regarding prognosis, the mSTESS score had statistical significance for poor outcomes (GOS ≤ 3) (95% CI, 1.45–4.00, *p *< 0.05).

**Conclusion:**

Following the occurrence of CSE, the duration of seizures is an independent risk factor for severity, while the mSTESS score has predictive significance for prognosis.

## Graphical abstract

The duration of seizures is an independent risk factor for the severity of CSE, and the mSTESS score has predictive significance for the prognosis of CSE.

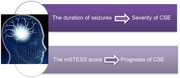

## 1 Introduction

Status epilepticus (SE) is a common critical neurological condition characterized by complex seizure types, with a high rate of disability and mortality. According to existing literature, the global annual incidence of SE is approximately 20–40 per 100,000 population, with mortality rates reaching 20%–30% (Mevius et al. 2023; Ascoli et al. 2021). A study focusing on Chinese patients reported SE mortality rates of approximately 10%–20% (Neligan and Rajakulendran 2024). Additionally, the incidence of SE in low‐ and middle‐income countries is generally higher than in high‐income countries, which may be attributed to the lack of medical resources, insufficient disease recognition, and inadequate treatment options (Singh and Gupta 2024). Among the different types of SE, convulsive status epilepticus (CSE) is the most acute and severe, accounting for approximately 45%–74% of all SE cases. It is characterized by sustained body rigidity, clonic movements, or continuous clonic activity, accompanied by altered consciousness. Comprehensive treatment is necessary, and it often leads to severe neurological deficits; the reported mortality rate for CSE ranges from 3.45% to 39% (Kämppi et al. 2015; Lv et al. 2017; Li et al. 2009). Therefore, clinical and basic research related to CSE has always been one of the hot and challenging issues in the field of neurological emergencies. Although the number of studies investigating risk factors for SE prognosis is continuously increasing, there are relatively fewer studies on CSE, and certain limitations persist. For instance, current research mainly targets pediatric or elderly populations, while studies on adult CSE patients remain limited (Aulická 2022; Yang et al. 2023; Boggs 2021). In this study, we analyze the clinical characteristics of CSE patients (> 15 years old) and explore risk factors associated with severity and outcomes to enable early prediction of treatment response and prognosis.

## 2 Material and Methods

### 2.1 Participants

We retrospectively gathered clinical data of CSE patients treated at Tianjin Huanhu Hospital from July 2023 to December 2024 by searching the medical records system. To maintain the security of patients' personal information, we anonymized key information that can identify patients after data extraction. Inclusion criteria: no restrictions on age or gender and meeting the diagnostic criteria for CSE as defined by the International League Against Epilepsy in 2015. This definition specifies that the seizure duration must exceed that of most similar seizures, without cessation, and consciousness must not return to baseline. We included cases of generalized tonic‐clonic seizures lasting more than 5 min, those with focal SE and consciousness impairment lasting more than 10 min, or patients who experienced two seizures within 5 to 30 min without full recovery of consciousness in between (Trinka et al. 2015). The exclusion criteria included non‐CSE patients and those with incomplete, unclear, or missing significant research data that could affect judgment. This study was approved by the Ethics Committee of Tianjin Huanhu Hospital (No. 2025–092).

### 2.2 Data Collection and Definitions

Data was collected using a structured questionnaire that recorded patients' demographic information, medical history, etiology, duration and frequency of seizures, treatment course, presence of pulmonary infection, uses of mechanical ventilation, and continuation of antiepileptic drugs (AEDs) at discharge. If the patient cannot complete the interview, the first choice of representatives is the patient's family who have lived together. Two experienced doctors independently assessed the patient's etiology using medical history and imaging examination and scored the modified Status Epilepticus Severity Score (mSTESS) at onset and the Glasgow Outcome Scale (GOS) at discharge. We then compared the two assessments. If there was disagreement, a third doctor evaluated the data independently. Finally, all three doctors discussed the case together to reach a consensus.

The mSTESS score was utilized to assess the severity of CSE, with a score of mSTESS ≥ 4 indicating severe illness and mSTESS < 4 indicating mild illness (González‐Cuevas et al. 2016). mSTESS is based on the STESS score combined with the modified Rankin Scale score (mRS), and it raises the cutoff age to 70 years, enhancing the predictive accuracy of the STESS score and reducing the prediction error for patients over 65 years old with no history of epilepsy. mSTESS improves the accuracy of assessing disease severity and is simple to implement, making it suitable for rapid assessment of CSE at onset (Yuan et al. 2018).

The GOS score could evaluate patients’ consciousness levels, self‐care abilities, and social functioning. Several previous studies have applied the GOS score to the prognostic evaluation of SE. Since a GOS score of ≤ 3 points indicates that the patient cannot live independently, we use this cutoff value to determine poor prognosis. Therefore, we utilized the GOS score for CSE prognostic evaluation, defining a favorable outcome as GOS > 3 and a poor outcome as GOS ≤ 3 (Jose et al. 2021; Krejzar et al. 2024; Kling et al. 2022).

### 2.3 Statistical Analysis

All statistical analyses were performed using R software 4.2.0. Which normally distributed continuous data were expressed as mean ± SD, and comparisons between two groups were made using a two‐sample *t*‐test. Categorical data were expressed as n (%) and analyzed using the chi‐square test or Fisher's exact test. Statistically significant indicators were included in binary multivariate logistic analysis. *p* < 0.05 was considered statistically significant.

## 3 Results

### 3.1 Clinical Characteristics

We searched the medical record system of Tianjin Huanhu Hospital from July 2023 to December 2024. Both automatic and manual methods were used with CSE, generalized SE, and epilepsy as search terms. A total of 312 patients were retrieved. Among them, 176 cases were non‐CSE, and 32 cases were excluded due to incomplete data recording. The study ultimately included the 104 CSE patients. Among them, 60 were male (57.7%) and 44 were female (42.3%). Among the participants, 76 individuals (73.1%) were diagnosed with GCSE. The average mSTESS score was 3.95 ± 1.50, with 65 patients (62.5%) scoring 4 or higher. The average duration of seizures was 2.07 ± 2.43 h. There were 33 individuals (31.7%) with a history of epilepsy, 57 individuals (54.8%) with a history of hypertension, 13 individuals (12.5%) with coronary heart disease, 47 individuals (45.2%) with stroke, 5 individuals (4.8%) with tumors, 5 individuals (4.8%) with traumatic brain injury, 21 individuals (20.2%) with diabetes, and 5 individuals (4.8%) with atrial fibrillation. During treatment, 60 patients (57.7%) developed pulmonary infections, 15 patients (14.4%) required mechanical ventilation, 8 patients (7.7%) progressed to refractory status epilepticus (RSE), and 2 individuals (1.9%) died during hospitalization. At discharge, the prognosis was poor, with GOS ≤ 3 for 40 patients (38.5%).

The most common clinical diagnosis was intracranial structural abnormalities, particularly strokes. The second most common cause was undetermined origins (21 cases, 20.2%), followed by intracranial infections (9 cases, 8.7%), autoimmune encephalitides (5 cases, 4.8%), and metabolic disorders (3 cases, 2.9%). All clinical characteristics are detailed in Table 1.

**TABLE 1:** The characteristics of the patients included in the study.

Patient demographics	
Total patients	104
Male/Female (%)	60(57.7%)/44(42.3%)
Median age, years (±SD)	58.05(±17.50)
Seizure type, GCSE, n (%)	76 (73.1%)
mSTESS score (±SD)	3.95 (±1.50)
mSTESS ≥ 4 (%)	65 (62.5%)
Duration of seizures, h (±SD)	2.07 (±2.43)
Medical history (%)	
Epilepsy	33 (31.7%)
Hypertension	57 (54.8%)
Diabetes	21 (20.2%)
Coronary heart disease	13 (12.5%)
Traumatic brain injury	5 (4.8%)
Atrial fibrillation	5 (4.8%)
Stroke	47 (45.2%)
Hemorrhagic stroke	11 (10.6%)
Ischemic stroke	40 (38.5%)
Tumor	5 (4.8%)
Reason for CSE (%)	
Abnormal brain structure	
Stroke	45 (43.3%)
Hemorrhagic stroke	15 (14.4%)
Ischemic stroke	29 (27.9%)
Subarachnoid hemorrhage	1 (1%)
ICVD	10 (9.6%)
Traumatic brain injury	5 (4.8%)
Vascular malformation	2 (1.9%)
Tumor	4 (3.8%)
3 (2.9%)	
Metabolic disease	5 (4.8%)
Autoimmune encephalitis	9 (8.7%)
Intracranial infection	21 (20.2%)
Undetermined	21 (20.2%)
Pulmonary infection (%)	60 (57.7%)
Mechanical ventilation (%)	15 (14.4%)
RSE (%)	8 (7.7%)
GOS score (±SD)	3.92 (±1.04)
GO S ≤3(%)	40 (38.5%)

### 3.2 Risk Factors for Severity of CSE

Based on the mSTESS score following the onset of CSE, patients were classified into a severe group (mSTESS ≥ 4) that included 65 individuals, representing 62.50%. Univariate analysis showed statistically significant differences between the two groups in several factors, including age (*p *= 0.012), seizure duration (*p *= 0.004), stroke (*p *= 0.046), hemorrhagic stroke (*p *= 0.001), history of stroke (*p *= 0.007), ischemic stroke history (*p *= 0.012), and hypertension history (*p *= 0.029). The data regarding the patients' severity are summarized in Table 2. When these influencing factors were included in a binary multivariate logistic regression model, the results indicated that the duration of seizures still had statistical significance regarding the severity of CSE (*p *= 0.027). The results of the multivariate logistic analysis concerning patient severity are presented in Table 3.

**TABLE 2:** Univariate analysis of CSE patients’ severity.

Variables	Total (*n* = 104)	mSTESS ≥ 4 (*n* = 65)	mSTESS < 4 (*n* = 39)	*p*
Age, Mean ± SD	58.05 ± 17.50	61.37 ± 16.47	52.51 ± 17.98	**0.012**
Gender, n(%)				0.151
Female	44 (42.31)	31 (47.69)	13 (33.33)	
Male	60 (57.69)	34 (52.31)	26 (66.67)	
Duration of seizures, mean ± SD	2.07 ± 2.43	2.50 ± 2.93	1.34 ± 0.88	**0.004**
Seizure type, GCSE, *n* (%)	75 (72.12)	48 (73.85)	27 (69.23)	0.611
Reason for CSE, *n* (%)				
Abnormal brain structure				
Stroke	45 (43.27)	12 (30.77)	33 (50.77)	**0.046**
Hemorrhagic stroke	15 (14.42)	15 (23.08)	0 (0.00)	**0.001**
Ischemic stroke	29 (27.88)	17 (26.15)	12 (30.77)	0.611
Subarachnoid hemorrhage	1 (0.96)	1 (1.54)	0 (0.00)	1.000
ICVD	10 (9.62)	6 (9.23)	4 (10.26)	1.000
Traumatic brain injury	5 (4.81)	2 (3.08)	3 (7.69)	0.554
Vascular malformation	2 (1.92)	1 (1.54)	1 (2.56)	1.000
Tumor	4 (3.85)	3 (4.62)	1 (2.56)	1.000
Metabolic disease	3 (2.88)	0 (0.00)	3 (7.69)	0.096
Autoimmune encephalitis	5 (4.81)	2 (3.08)	3 (7.69)	0.554
Intracranial infection	9 (8.65)	5 (7.69)	4 (10.26)	0.928
Undetermined	21 (20.19)	14 (21.54)	7 (17.95)	0.659
Medical history, n(%)				
Epilepsy	33 (31.73)	20 (30.77)	13 (33.33)	0.786
Hypertension	57 (54.81)	41 (63.08)	16 (41.03)	**0.029**
Diabetes	21 (20.19)	17 (26.15)	4 (10.26)	0.051
Coronary heart disease	13 (12.50)	8 (12.31)	5 (12.82)	1.000
Traumatic brain injury	5 (4.81)	3 (4.62)	2 (5.13)	1.000
Atrial fibrillation	5 (4.81)	3 (4.62)	2 (5.13)	1.000
Stroke	47 (45.19)	11 (28.21)	36 (55.38)	**0.007**
Hemorrhagic stroke	11 (10.58)	9 (13.85)	2 (5.13)	0.285
Ischemic stroke	40 (38.46)	31 (47.69)	9 (23.08)	**0.012**
Tumor	5 (4.81)	3 (4.62)	2 (5.13)	1.000

The bold values indicate that the values are statistically significant (*p* < 0.05).

**TABLE 3:** Multivariate logistic analysis of the severity of CSE patients.

Variables	*β*	SE	*Z*	*p*	OR (95% CI)
Age	0.01	0.02	0.87	0.384	1.01 (0.98–1.04)
Duration of seizures	0.48	0.22	2.21	**0.027**	1.61 (1.06–2.47)
Reason for CSE					
Stroke	0.11	0.54	0.20	0.845	1.11 (0.38–3.21)
Hemorrhagic stroke	35.38	3234.01	0.01	0.991	2,328,501,186,507,475.00 (0.00–Inf)
Medical history					
Hypertension	0.26	0.56	0.46	0.642	1.30 (0.43–3.92)
Stroke	−17.44	2352.87	−0.01	0.994	0.00 (0.00–Inf)
Ischemic stroke	18.42	2352.87	0.01	0.994	100,048,150.08 (0.00–Inf)

Abbreviations: CI, confidence interval; OR, odds ratio.

The bold values indicate that the values are statistically significant (*p* < 0.05).

### 3.3 Risk Factors on CSE Outcomes

Patients were categorized according to their GOS score at discharge, with 40 individuals classified as having a poor prognosis (GOS ≤ 3), representing 38.46%. Univariate analysis revealed statistically significant differences between the two groups in several factors, including age (*p* < 0.001), duration of seizures (*p *= 0.039), mSTESS score (*p* < 0.001), hemorrhagic stroke (*p *= 0.015), history of hypertension (*p *= 0.004), history of stroke (*p* < 0.001), history of hemorrhagic stroke (*p *= 0.032), history of ischemic stroke (*p *= 0.006), and pulmonary infection (*p *= 0.001). Outcome data for the patients are summarized in Table 4. When these influencing factors were included in a binary multivariate logistic regression model, the results indicated that the mSTESS score remained statistically significant regarding poor outcomes (*p* < 0.001). Multivariate logistic analysis data on the prognosis of patients are shown in Table 5.

**TABLE 4:** Univariate analysis of prognosis in CSE patients.

Variables	Total (*n* = 104)	GOS > 3 (*n* = 64)	GOS ≤ 3 (*n* = 40)	*p*
Age, mean ± SD	58.05 ± 17.50	53.69 ± 19.34	65.03 ± 11.13	**< 0.001**
Gender, *n* (%)				0.975
Female	44 (42.31)	27 (42.19)	17 (42.50)	
Male	60 (57.69)	37 (57.81)	23 (57.50)	
Duration of seizures, mean ± SD	2.07 ± 2.43	1.68 ± 1.86	2.69 ± 3.07	**0.039**
Seizure type, GCSE, *n* (%)	75 (72.12)	44 (68.75)	31 (77.50)	0.333
Frequency, mean ± SD	5.62 ± 5.07	5.39 ± 5.48	5.97 ± 4.38	0.570
mSTESS score, mean ± SD	3.95 ± 1.50	3.33 ± 1.35	4.95 ± 1.15	**< 0.001**
Reason for CSE, *n* (%)				
Abnormal brain structure				
Stroke	45 (43.27)	25 (39.06)	20 (50.00)	0.273
Hemorrhagic stroke	15 (14.42)	5 (7.81)	10 (25.00)	**0.015**
Ischemic stroke	29 (27.88)	19 (29.69)	10 (25.00)	0.604
Subarachnoid hemorrhage	1 (0.96)	1 (1.56)	0 (0.00)	1.000
ICVD	10 (9.62)	5 (7.81)	5 (12.50)	0.655
Traumatic brain injury	5 (4.81)	2 (3.12)	3 (7.50)	0.587
Vascular malformation	2 (1.92)	1 (1.56)	1 (2.50)	1.000
Tumor	4 (3.85)	1 (1.56)	3 (7.50)	0.314
Metabolic disease	3 (2.88)	3 (4.69)	0 (0.00)	0.431
Autoimmune encephalitis	5 (4.81)	3 (4.69)	2 (5.00)	1.000
Intracranial infection	9 (8.65)	8 (12.50)	1 (2.50)	0.160
Undetermined	21 (20.19)	13 (20.31)	8 (20.00)	0.969
Medical history, *n* (%)				
Epilepsy	33 (31.73)	21 (32.81)	12 (30.00)	0.764
Hypertension	57 (54.81)	28 (43.75)	29 (72.50)	**0.004**
Diabetes	21 (20.19)	11 (17.19)	10 (25.00)	0.334
Coronary heart disease	13 (12.50)	6 (9.38)	7 (17.50)	0.223
Traumatic brain injury	5 (4.81)	2 (3.12)	3 (7.50)	0.587
Atrial fibrillation	5 (4.81)	2 (3.12)	3 (7.50)	0.587
Stroke	47 (45.19)	20 (31.25)	27 (67.50)	**< 0.001**
Hemorrhagic stroke	11 (10.58)	3 (4.69)	8 (20.00)	**0.032**
Ischemic stroke	40 (38.46)	18 (28.12)	22 (55.00)	**0.006**
Tumor	5 (4.81)	2 (3.12)	3 (7.50)	0.587
RSE, *n* (%)	8 (7.69)	3 (4.69)	5 (12.50)	0.282
Pulmonary infection, *n* (%)	60 (57.69)	29 (45.31)	31 (77.50)	**0.001**
Mechanical ventilation, *n* (%)	15 (14.42)	6 (9.38)	9 (22.50)	0.064

The bold values indicate that the values are statistically significant (*p* < 0.05).

**TABLE 5:** Multivariate logistic analysis of prognosis in CSE patients.

Variables	*β*	SE	*Z*	*p*	OR (95% CI)
Age	0.01	0.02	0.70	0.483	1.01 (0.98–1.05)
Duration of seizures	0.09	0.11	0.83	0.406	1.10 (0.88–1.37)
mSTESS score	0.88	0.26	3.41	**< 0.001**	2.41 (1.45–4.00)
Reason for CSE					
Hemorrhagic stroke	−0.71	0.83	−0.86	0.392	0.49 (0.10–2.51)
Pulmonary infection	1.13	0.61	1.84	0.066	3.09 (0.93–10.28)
Medical history					
Stroke	2.96	2.04	1.45	0.147	19.27 (0.35–1048.28)
Hemorrhagic stroke	0.28	1.66	0.17	0.865	1.33 (0.05–34.67)
Ischemic stroke	−1.92	1.94	−0.99	0.322	0.15 (0.00–6.57)
Hypertension	0.84	0.60	1.40	0.162	2.31 (0.71–7.51)

Abbreviations: CI, confidence interval; OR, odds ratio.

The bold values indicate that the values are statistically significant (*p* < 0.05).

## 4 Discussion

Our study demonstrated that among CSE patients, males outnumbered females, which aligns with previous reports (Neligan and Rajakulendran 2024). In this cohort, GCSE accounted for 76% of cases, higher than the historically reported proportion of 30%–50% in all CSE cases. This discrepancy may be closely associated with variations in patients’ clinical characteristics, underlying diseases, and treatment responses (Aulická 2022). Notably, the proportion of GCSE may be even higher in intensive care unit (ICU) settings (Parikh et al. 2019).

The predominant etiology of CSE was structural brain abnormalities, followed by unknown causes, intracranial infections, autoimmune encephalitis, and metabolic disorders. Among structural abnormalities, stroke—including hemorrhagic, ischemic, and subarachnoid hemorrhage—constituted the largest proportion, consistent with prior studies identifying cerebrovascular disease as the leading cause of CSE (Zhang et al. 2019). Moreover, compared to developed countries such as the United States, China exhibits a higher incidence of cerebrovascular diseases (Ashraf et al. 2025). In stroke patients, particularly those with large hemispheric infarctions or hemorrhages (> 30 mL), the incidence of SE may reach 20%–30% (Sun et al. 2024). In this study, post‐stroke CSE cases included nine patients with large hemispheric infarctions and six with cerebral hemorrhages exceeding 30 mL.

We used the mSTESS score to evaluate the severity of CSE in this study. Through multifactorial logistic analysis, we found that the duration of CSE is an independent risk factor affecting its severity. This is because the duration of CSE closely correlates with the extent of neuronal injury. Research indicates that CSE leads to acute neuronal damage and long‐term functional impairment; the longer the seizures last, the more severe the damage becomes. This process primarily occurs due to excitotoxicity and metabolic imbalances. Excitotoxicity refers to the excessive stimulation of neurons by excitatory neurotransmitters (like glutamate) during a seizure, leading to excessive calcium ions flowing into the cells and subsequently triggering cell death (Medzhitov 2021). In addition, SE may also exacerbate oxidative stress and inflammatory responses, and these factors work together to further aggravate neuronal damage and death, resulting in more severe conditions and poor prognosis (Shefler et al. 2022). Therefore, when assessing that a patient's CSE condition is relatively mild, early intensified antiepileptic treatment (such as a drug‐induced coma) may not be considered (Rossetti et al. 2008).

Many studies assessing prognosis in CSE have used patient mortality as an indicator of poor outcomes. However, in this study, two patients died in the hospital, while four patients were discharged by their families when their condition changed. Given the small sample size, we believed that selecting mortality as the outcome measure might overestimate patient prognosis. During follow‐up, some relatives were unable to provide accurate information about the patients' mortality due to time intervals. This lack of information hindered our ability to determine whether the patients' deaths were directly related to CSE. Therefore, we did not adopt mortality as the prognostic indicator. We referred to some previous studies and selected the GOS score as an indicator for evaluating prognosis. Our multifactorial logistic analysis indicated that mSTESS is an independent risk factor for poor prognosis in CSE, consistent with previous research that suggests mSTESS can more accurately predict outcomes for SE patients (González‐Cuevas et al. 2016; Yuan et al. 2018).

Jie Zhang et al. found that cerebrovascular diseases are the primary causes of SE and a significant contributor to mortality. This is consistent with our study results that cerebrovascular diseases are the main cause of CSE. However, our multifactorial analysis did not reveal a correlation between etiology with severity and short‐term prognosis. This lack of correlation may result from the broad classification of causes and a limited number of cases (Jose et al. 2021). Hay et al. reported that 20%–65% of CSE patients develop infections during seizures, and the poor prognosis and mortality rate of patients with CSE receiving mechanical ventilation significantly increase (Krejzar et al. 2024). In this study, the incidence of lung infections after CSE was 57.7%, and mechanical ventilation was 14.4%, which is generally consistent with previous studies. However, in our study, we found no clear correlation between the pulmonary infection and mechanical ventilation with poor outcomes, which may be due to the small sample size and the differing outcome indicators selected.

## 5 Limitations

Our study has several limitations. First, as a single‐center study with a small sample size, our results may be biased. Second, being a retrospective study, we encountered incomplete EEG (electroencephalogram) data for some patients, preventing its analysis, despite EEG being a crucial indicator for determining the etiology and efficacy of CSE. Additionally, tracing the medication history of epilepsy patients before admission was challenging; some CSE episodes may relate to missed doses or insufficient dosages of AEDs, which we could not address in this study. Third, the evaluation content we chose lacks relevant data on biomarkers and imaging, which may provide certain guidance for predicting the prognosis of CSE. Future studies can expand the sample size and conduct multicenter prospective research for a more in‐depth exploration of the CSE population.

## 6 Conclusion

Our study confirms previous findings that stroke is the main cause of CSE. The duration of CSE is directly related to the severity of its onset. Specifically, longer seizure durations lead to more severe CSE conditions. Additionally, the mSTESS score serves as a risk factor for prognosis following CSE; higher scores indicate greater chances of consciousness impairment and reduced ability to live independently upon discharge. Timely and accurate assessment of the patient's condition and prognosis after CSE is crucial for choosing effective treatment plans.

Thus, clinicians should monitor seizure duration and mSTESS scores early in the course of the disease to enhance outcomes for CSE patients.

## Author Contributions

**Zhen Tian**: conceptualization, formal analysis, writing – original draft, writing – review and editing. **Yingchun Xu**: formal analysis, data curation, conceptualization. **Rundong He**: software, data curation. **Di Huang**: software, data curation. **Wanyu Liu**: validation. **Peiwen Li**: validation. **Jiasheng Yang**: data curation. **Jie Yang**: data curation. **Jing Ding**: supervision, resources. **Wei Yue**: funding acquisition, supervision, writing – review and editing.

## Ethics Statement

This study was approved by the Ethics Committee of Tianjin Huanhu Hospital (No. 2025–092). Given the retrospective nature of the study, the necessity for written informed consent was waived.

## Conflicts of Interest

The authors declare no conflicts of interest.

## Peer Review

The peer review history for this article is available at https://publons.com/publon/10.1002/brb3.70681

## Data Availability

The data used to support the findings of this study are available from the corresponding author upon request (hhyuewei2021@163.com).
